# Field evaluation of a mobile biosafety laboratory in Senegal to strengthen rapid disease outbreak response and monitoring

**DOI:** 10.4102/ajlm.v9i2.1041

**Published:** 2020-08-20

**Authors:** Cheikh Fall, Aurélie Cappuyns, Oumar Faye, Steven Pauwels, Gamou Fall, Ndongo Dia, Moussa M. Diagne, Cheikh T. Diagne, Makhtar Niang, Alassane Mbengue, Martin Faye, Idrissa Dieng, Babacar Gningue, Abdoulaye Bousso, Ousmane Faye, Rudi Pauwels, Amadou A. Sall

**Affiliations:** 1 Department of Virology, Institut Pasteur de Dakar, Dakar, Senegal; 2 Praesens Foundation, Brussels, Belgium; 3 Quality Department, Institut Pasteur de Dakar, Dakar, Senegal; 4 Senegalese Health Emergency Operation Center, Ministry of Health, Dakar, Senegal

**Keywords:** mobile biosafety laboratory, MBS-Lab, field deployment, outbreak, Senegal, point-of-care

## Abstract

**Background:**

Past and recent outbreaks have highlighted the vulnerability of humans to infectious diseases, which represent serious economic and health security threats. A paradigm shift in the management of sanitary crises is urgently needed. Based on lessons from the 2014 Ebola outbreak, the Praesens Foundation has developed an all-terrain mobile biosafety laboratory (MBS-Lab) for effective field diagnostics capabilities.

**Objective:**

The aim of the study was to train African teams and run a field evaluation of the MBS-Lab, including robustness, technical and operational sustainability, biosafety, connectivity, turn-around times for testing and result delivery.

**Methods:**

The MBS-Lab was deployed in Senegal in October 2017 for a six-month field assessment under various ecological conditions and was mobilised during the dengue outbreaks in 2017 and 2018.

**Results:**

The MBS-Lab can be considered an off-grid solution that addresses field challenges with regard to working conditions, mobility, deployment, environment and personnel safety. Blood (*n* = 398) and nasal swab (*n* = 113) samples were collected from 460 study participants for molecular screening for acute febrile illnesses and respiratory infections. The results showed that malaria (particularly in Kédougou) and upper respiratory tract infections remain problematic. Suspected dengue samples were tested on board during the dengue outbreaks in 2017 (882 tests; 128 confirmed cases) and 2018 (1736 tests; 202 confirmed cases).

**Conclusion:**

The MBS-Lab is an innovative solution for outbreak response, even in remote areas. The study demonstrated successful local ownership and community engagement. The MBS-Lab can also be considered an open mobile healthcare platform that offers various opportunities for field-deployable, point-of-care technologies for surveillance programmes.

## Introduction

Past and recent disease outbreaks (e.g. severe acute respiratory syndrome, Middle East respiratory syndrome-related coronavirus, severe acute respiratory syndrome coronavirus, Ebola, Zika, dengue) have shown that infectious diseases continue to affect the lives of people, while also representing social, economic and national security threats that can quickly evolve into global health crises.^1^ For example, the 2014–2016 Ebola outbreak in West Africa cost $32.6 billion and resulted in the loss of 11 000 lives.^2^ Furthermore, dengue fever has become endemic in Africa with recurrent outbreaks in different countries.^3,4,5^ A parallel threat is the rise of antimicrobial resistance with an estimated 4.1 million deaths per year, expected to lead to a $42 trillion loss to the African economy by 2050.^2^ It is therefore urgent to further strengthen infectious disease surveillance and outbreak preparedness. Compared to the resources devoted to dealing with other global threats such as terrorism, climate change or war, little investment is dedicated to infectious disease outbreak preparedness.^6,7,8^ In recent years, health professionals and decision-makers have identified several strategies needed to mitigate risk.^9^ However, up to now, preventive initiatives have underestimated threats. In view of this, there is an increasing international community alliance, not only to raise funding, but more importantly to develop adequate and rapid deployment of task forces to prevent or contain declared outbreaks.^10^

In fact, the typical pattern of infectious disease preparedness today can be characterised by international mobilisation during outbreaks, followed by relaxation and diminishing investments. The dependence on crisis response is both costly and ineffective (especially in preventing future outbreaks).^8^ Whereas ignorance and lack of technology may have been an excuse in the past, more can and should be done for the development of strategies to achieve global health security, including a commitment from public authorities, the availability of appropriate healthcare infrastructure and qualified staff, and international operating ‘disease-fighting forces’ that can be deployed rapidly when needed.^11,12^ According to the World Bank, Africa needs between $2 billion and $3.5 billion a year for epidemic preparedness. In addition, this requires political leadership, financial commitment, partnerships and innovation.^11^

Based on their field observations in West Africa during the 2014–2016 Ebola epidemic and driven by the ambition to bring advanced technologies to communities that need it most, the Praesens Foundation developed a truck-based mobile biosafety laboratory (MBS-Lab) that included an isolator for safe handling of samples and a fully automated molecular diagnostic platform. The goal was to develop an additional tool for better preparedness and faster response to outbreaks and epidemics. The purpose was to fill the gap between the so-called suitcase-based or boxed field mobile laboratories and the much larger container-based laboratories. This combines rapidity, biosafety and advanced diagnostic technology for the rapid detection and identification of pathogens, even in hard-to-reach regions with limited to non-existent healthcare infrastructure.

The key objective of this study was to evaluate the MBS-Lab in an African context and to prepare local staff for its use, especially during outbreaks. The MBS-Lab was challenged under various field conditions to test its operational readiness regarding the following indicators: robustness, technical and operational sustainability, autonomy, connectivity, maintenance, biosafety procedures, logistics, turn-around times and communication of test results. With its current ecosystem of fixed laboratories, field stations, national surveillance network and experience in responding to various outbreaks, the Institut Pasteur de Dakar (IPD), which hosts a World Health Organization Collaborating Center for arboviruses and haemorrhagic fever viruses, was chosen as a partner to evaluate this MBS-Lab.

## Methods

### Ethical considerations

Ethical approval to conduct the study was provided by the Ministry of Health, Republic of Senegal (0154/MSAS/DPRS/CNERS; Protocol SEN17/48).

### Biosafety considerations

A closed biosafety isolator equipped with a high-efficiency particulate air filter was integrated in the MBS-Lab. A negative pressure cascade (−25 pascal [Pa], −50 Pa and −25 Pa inside the isolator) provided a safe environment for handling different classes of pathogen, thereby eliminating the need for personal protective equipment required for high-containment laboratory facilities.

In addition, regular decontaminations were conducted by fumigation with hydrogen peroxide (H_2_O_2_) both within the isolator and the workspace of the MBS-Lab (Nocospray, Oxy’Pharm, Paris, France). To prevent external contamination and ensure cleanliness inside the MBS-Lab, operators were required to wear disposable shoe covers.

### Study setting

**FIGURE 1:**
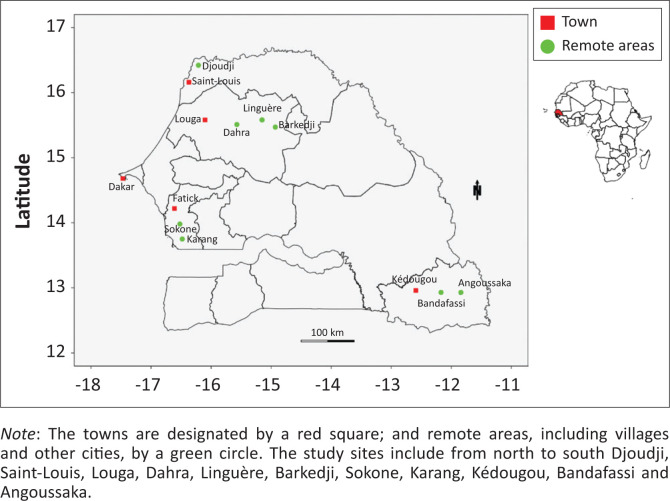
Map indicating the study areas in Senegal, 2017–2018.

The MBS-Lab was built in Belgium and shipped to Dakar, Senegal, in September 2017. This prospective study was carried out in five different localities in Senegal based on their climatological and ecological differences (from the dry northern to the humid southern regions) for six months (from October 2017 to March 2018). Work was conducted in various healthcare settings ranging from district hospitals and laboratories to primary healthcare settings and outreach initiatives in remote areas. After a week of training of IPD staff, the MBS-Lab was first deployed in the Kédougou area (south-eastern Senegal) from 08 to 23 October 2017, before being mobilised unexpectedly for dengue outbreak management in Louga city (north-western Senegal) from 25 October to 23 November 2017. Following this event, extensive field evaluation continued in the following sites: (1) Barkedji, Linguère and Dahra localities, which are not far from Louga (03–29 December 2017), (2) Bandafassi and Angoussaka villages near Kédougou (13–27 January 2018), (3) Saint-Louis city and Debi-Tiguette village next to the bird sanctuary of Djoudj in the north (11–23 February 2018) and (4) Sokone and Karang in the Fatick region in the central part of country, near The Gambia (11–24 March 2018) (Figure 1). The MBS-Lab travelled over 7000 kilometres during the pilot study.

Prior to deployment, IPD laboratory staff received appropriate training to work in the MBS-Lab, which covered (1) necessary precautions to prevent exposures, (2) biosafety practices and procedures and (3) data and material management. In addition, the training covered realistic scientific, medical, technical and operational challenges that could be encountered in a field situation. Drivers and maintenance technicians were also trained extensively for their jobs. Overall, a team of more than 15 people have been trained and have participated in one or more field deployments.

### Study population and sampling

The study population included people visiting local healthcare facilities and presenting acute febrile illness or respiratory symptoms. They consisted of male and female patients of all ages who fully consented to participate in the study. For children and patients under 18 years of age, the parents or legal guardians signed the consent forms.

A syndromic approach was adopted for diagnostics. Therefore, blood (*n* = 398) and nasopharyngeal swab (*n* = 113) samples were collected and analysed using a multiplex strategy for the detection of *Plasmodium* genus, arboviruses (including main flavivirus species, chikungunya and Rift Valley fever virus), *Salmonella* genus and respiratory viruses (such as influenza, respiratory syncytial virus and human metapneumovirus).

### Staff composition

One scientist was in charge of coordination between local healthcare settings and the laboratory, such as specimen reception and the release of analysis results. Two laboratory technicians were in charge of operations, including molecular diagnostic activities, cleaning of the laboratory and daily reporting. The dedicated driver of the MBS-Lab assisted in setting up the laboratory and stabilising the vehicle. During operations, he ensured security around the laboratory and monitored its energy supply. He was also trained to resolve any minor technical issues related to the vehicle and equipment. Maintenance and biosafety personnel were on call in Dakar and assisted in the field on request.

### Mobile biosafety laboratory workflow

#### Sample reception

Samples and clinical information forms were collected daily on-site and from neighbouring healthcare facilities for analysis in the MBS-Lab, respecting cold-chain protocols during transportation. Samples were then introduced from the outside directly into the isolator using the secured exterior sample hatch. The access to the sample hatch and entrance to the MBS-Lab were for authorised personnel only, using a magnetic badge or key.

#### Handling inside the isolator

**FIGURE 2:**
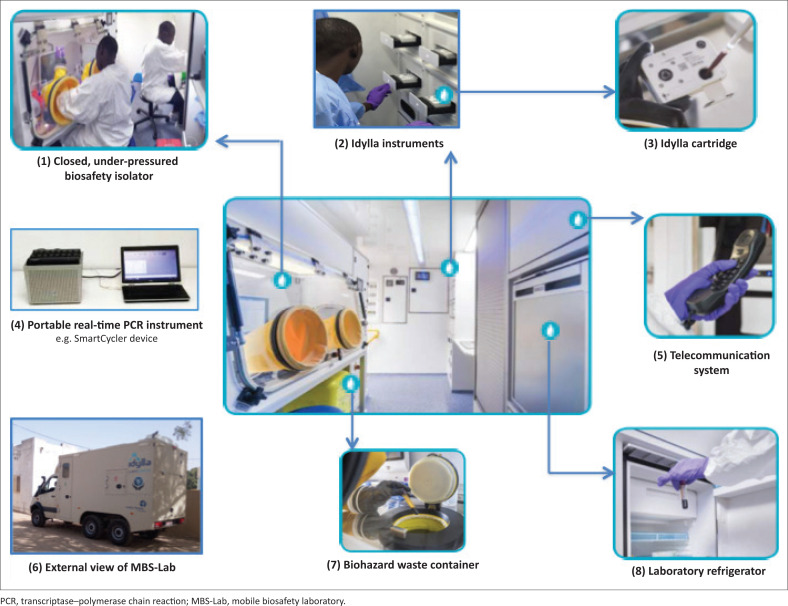
Features of the mobile biosafety laboratory and platforms, Senegal, 2017–2018. (1) Closed under-pressured biosafety isolator; (2) Idylla platform; (3) Idylla cartridge; (4) SmartCycler instrument; (5) Telecommunication system; (6) External view of the MBS-Lab; (7) Biohazard waste container; (8) Laboratory refrigerator.

For the protection of laboratory staff and the environment, all samples were unpacked only inside the isolator, in order to minimise the risk of exposure (Figure 2). The surfaces of packaging and sample tubes were decontaminated with Aniospray disinfectant (Laboratoires ANIOS, Lille, France). Easy and safe handling was the main objective for the design of the isolator with a negative internal pressure of up to −50 Pa. The entrance and exit pass boxes flanking the isolator were set at −25 Pa. Therefore, specimens containing any class of infectious pathogen can be handled within the MBS-Lab.

#### Molecular platforms

The modularity and flexibility of the MBS-Lab enable rapid detection and identification of various pathogens using the on-board multiplexing molecular platforms.

The Idylla™ system (Biocartis, Mechelen, Belgium) is a fully automated, real-time polymerase chain reaction (real-time transcriptase–polymerase chain reaction) molecular testing system designed to offer results in the minimum amount of time. All components required for nucleic acid extraction, purification, real-time transcriptase–polymerase chain reaction amplification and detection are integrated in a single cartridge that was further loaded into the Idylla^TM^ system. Handling time was less than five min per sample and the liquid-tight, disposable cartridges greatly reduce the risk of contamination.^13^ The Idylla™ Ebola virus Triage Test, which was approved by the United States Food and Drug Administration for emergency use authorisation, and Tropical Fever Panel cartridges (prototype assay) were tested and compared with reference methods (manuscript in progress).^14^

In addition, real-time multiplex transcriptase–polymerase chain reaction tests were performed on the SmartCycler device (Cepheid, Sunnyvale, California, United States), using a set of LightMix kits containing pre-mixed primers and probes (TIB Molbiol, Berlin, Germany) for the simultaneous detection and differentiation of up to three different pathogens in less than 1 h. Results were analysed according to the manufacturer’s recommendations. Runs were valid if results generated for all controls (positive and negative) were correct. Samples were considered positive if there was an amplification curve with a crossing point value within the defined cut-off (crossing point < 39), equivocal if the crossing point value was higher than the cut-off and negative if there was no amplification.

#### Specimen and reagents storage

The MBS-Lab was equipped with a 4 °C refrigerator for short-term storage of inactivated products and reagents (Figure 2). In addition, there was a portable ultra-low mini −80 °C freezer (Shuttle™ ULT-25NE, Stirling, Athens, Ohio, United States) with capacity to store up to 1000 specimens, which were first stowed in cryoboxes.

#### Testing report, data processing and analysis

Prior to testing, samples were codified with unique anonymous numbers for traceability purposes, linking the sample number, patient identification, case definition and consent form. Data were reported in a Microsoft Excel database (Microsoft Corporation, Redmond, Washington, United States), cleaned, and analysed using R software (R Foundation, Vienna, Austria). The diagnostic results were delivered to physicians as early as possible for improved patient management.

## Results

Overall, 460 participants, consisting of 224 women and 236 men (sex ratio = 1.05), were recruited. The median age was 18 years (range: 2 months to 70 years). A total of 398 blood samples and 113 nasal swabs were screened using the commercial LightMix kits (TIB Molbiol, Berlin, Germany) based on a syndromic approach for the diagnosis of febrile infections, including malaria, those caused by *Salmonella* or arboviruses (chikungunya and Rift Valley fever virus, and flaviviruses such as dengue, Zika, West Nile and yellow fever virus) and respiratory diseases associated with influenza, parainfluenza, human metapneumovirus, adenovirus and respiratory syncytial viruses.

The results showed that malaria remains problematic in Senegal, particularly in the southern areas, where 60% (98/162) of the blood samples collected in Kédougou were positive (Table 1). The other malaria cases (15%, 29/195) were reported during the deployment in the north-western areas (Barkedji and Dahra-Linguère) in December 2017.

**TABLE 1:** Distribution of blood samples collected and pathogens identified in Kédougou, Barkedji, Dahra-Linguère, Saint-Louis and Karang-Sokone areas, October 2017–March 2018.

Site	Pathogens						Total
	Malaria	Flavi	ChikV	RVFV	Salmonella	Neg	
Kédougou 1	81	0	0	0	0	8	89
Barkedji	16	1	0	0	0	79	96
Dahra-Linguère	13	2	0	0	0	84	99
Kédougou 2	19	0	0	0	0	56	75
Saint-Louis	0	0	0	0	0	8	8
Sokone-Karang	0	0	0	0	0	33	33

**Total**	**129**	**3**	**0**	**0**	**0**	**268**	**400**

RVFV, Rift Valley fever virus; ChikV, chikungunya virus; Flavi, flavivirus; Neg, negative samples.

The other major health problem was respiratory tract infections, particularly among young children. Indeed, 21 metapneumovirus, 15 influenza, 3 parainfluenza and 4 picornavirus cases were diagnosed from nasal swab samples. Co-infection was found in two patients with metapneumovirus and influenza virus, one with malaria and metapneumovirus, one with malaria and picornavirus and one with metapneumovirus and picornavirus (Tables 1 and 2).

**TABLE 2:** Distribution of nasopharyngeal swab samples collected and pathogens identified in Kédougou, Barkedji, Dahra-Linguère, Saint-Louis and Karang-Sokone areas, October 2017–March 2018.

Site	Pathogens						Total
	Para-Inf	InfA	InfB	MPV	PicoV	Neg	
Kédougou 1	0	1	1	0	0	4	6
Barkedji	0	1	1	1	0	16	19
Dahra-Linguère	0	7	3	2	1	4	17
Kédougou 2	2	0	0	8	2	31	43
Saint-Louis	1	3	0	14	2	6	26
Sokone-Karang	0	0	0	0	1	10	11

**Total**	**3**	**12**	**5**	**25**	**6**	**71**	**122**

Para-Inf, parainfluenza virus; InfA, influenza A virus; InfB, influenza B virus; MPV, metapneumovirus; PicoV, picornavirus; Neg, negative samples.

Otherwise, the MBS-Lab was mobilised during the 2017 and 2018 dengue outbreaks at the request of the Ministry of Health. Consequently, 882 and 1736 suspected samples have been tested on-board, with 128 and 202 confirmed cases (manuscript in progress).

### Performance of the MBS-Lab

The features and performance of the MBS-Lab are detailed below.

#### All-terrain truck

**FIGURE 3:**
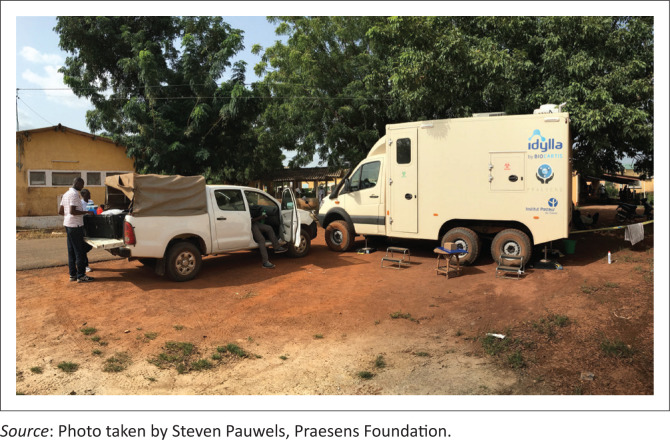
Deployment and implementation of the mobile biosafety laboratory in the field, Kédougou, Senegal, October 2017.

A Mercedes Sprinter was converted with a six-wheel driveline and increased gross vehicle mass to 7 tons. A fixed laboratory cabin was then installed on the chassis. This combination offers a good balance between optimised mobility, size and robustness. The MBS-Lab fills the gap between the extreme flexibility and mobility of suitcase-based mobile laboratories and fixed container-based solutions. The all-terrain vehicle can cover most of Africa’s diverse terrain and poor roads with a driving range of about 550 km. A support vehicle (Toyota Hilux) accompanied the MBS-Lab during deployments for personnel transportation and for carrying additional laboratory materials (Figure 3).

#### Power system

Power for the MBS-Lab was supplied by lithium-ion batteries, which can be charged by the on-board diesel generator, the local electrical grid (with an inline voltage regulator) or by the vehicle’s alternator. This system guaranteed energy autonomy for the proper functioning of the fully equipped laboratory, including the air conditioning, refrigerator, lighting, telecommunication system and diagnostic equipment (Idylla™ and SmartCycler™ instruments) for at least one working day relying on batteries alone. The whole laboratory was self-reliant for at least a week; the air conditioning system had the largest impact on energy consumption. The vehicle was equipped with a 100-litre fuel tank and as long as diesel fuel is available, the laboratory can be operational. In the event of a total power cut-off, the isolator had a dedicated uninterruptible power supply to ensure safe shut down and decontamination. The entire power management system can be remotely monitored and controlled using the on-board communication capabilities.

#### Geolocation system

A real-time satellite geo-positioning system was installed that allows for remote tracking of the vehicle. The system sent alerts when the MBS-Lab left or entered a predefined geofenced zone. The cabin was equipped with an emergency distress signal and badge identification of drivers for security and traceability.

#### Communication system

The MBS-Lab was equipped with 3G/4G cellular routers and worldwide-secured satellite networks, which guaranteed permanent connectivity and secure communication channels between the on-field mobile laboratory and the external world. Both systems also had the ability to create a local Wi-Fi network. These allowed for the remote monitoring of connected instruments and control computers that together form a data acquisition and monitoring system. The status and data of the MBS-Lab, including the isolator pressures, local time, temperature, humidity, power system monitoring and control, test reports and error logs, were displayed on several dedicated screens in the MBS-Lab.

#### Laboratory air conditioning

The MBS-Lab interior was kept at a controlled temperature and humidity even in hot (up to 54 °C) and humid environments. This not only created a comfortable working environment for personnel during long shifts in the laboratory, but also ensured that all equipment could function within optimal operating conditions.

#### Laboratory refrigerator

A laboratory refrigerator (4 °C) provided a cold chain for the short storage of reagents as well as (inactivated) samples for further confirmation testing. The safe storage of vaccines and medicines and their transportation to and from faraway health clinics until the point of administration is another potential use.

#### Hydraulic levelling system

This system automatically stabilises and levels the MBS-Lab in less than five minutes on uneven terrain. Once deployed, the MBS-Lab is no longer susceptible to any motion induced by people entering the laboratory or by wind shear, and complies with the requirements of certain equipment to only be operated when levelled.

#### Maintenance

Because the base vehicle was kept standard and is based on a widely used light commercial vehicle, the manufacturer’s recommended engine maintenance intervals are not affected and regular spare parts can be used. A selection of spare parts for both the vehicle and the power supply equipment is always on board for field repairs. Special spare parts were shipped to Dakar when needed and installed when the MBS-Lab was between deployments. In one instance, a maintenance technician was sent to the field to perform an urgent repair. The power supply system can be monitored and controlled remotely, which allows the system to send out alerts in case of issues that can also be resolved remotely, sometimes in combination with the installation of spare parts carried on board such as fuses. Systematic technical checks were performed in Dakar after each mission to keep the MBS-Lab ready for its next (unexpected) deployment.

#### Turn-around times

Prior to the availability of the MBS-Lab, samples had to be sent to IPD for specific diagnostic testing, causing slow turn-around times, which have a considerable effect on the quality of samples, the reliability of tests and the reporting of results. The decentralisation of testing with the MBS-Lab has dramatically cut turn-around times for samples processing and results delivery from at least a week to hours (average: 4 h).

#### Biosafety risk management

The MBS-Lab was designed for the safe handling of all types of pathogens and reduced human and environmental exposure to potentially harmful agents. The three key elements of biological containment are laboratory practices, safety equipment and facility design. Some characteristics are detailed below.

#### Personnel flow

Usually the MBS-Lab’s occupancy does not exceed two laboratory technicians. Before and after manipulation, the surface and materials used were decontaminated with Aniospray disinfectant (Laboratoires ANIOS, Lille, France).

#### Samples and materials flow

Samples and materials were brought into the isolator through the first pass box with a negative pressure of −25 Pa. This pass box has two entry doors, one inside the laboratory for materials and one connected to the external sample hatch. Accordingly, samples were deposited from outside into the MBS-Lab via this sample hatch and from there were handled safely within the isolator, avoiding any contact between the sample and the laboratory technician. A unidirectional workflow was respected.

#### Waste management

All potentially infectious waste materials were disposed of in a leak-proof biohazard waste container (JCE Biotechnology, ZA Bioparc, Lyon, France) (compliant with the normative requirements NF X 30-511, UN 3291 and UN 3249). This was safely connected to the inside of the isolator via a leak-proof hatch system (according to the recommendations of the International Organisation for Standardization [ISO] 10648-2), preventing contamination risks.^15,16^ Any materials which had to be taken out of the isolator, including RNA samples and test cartridges, were disinfected with Aniospray (Laboratoires ANIOS, Lille, France) and placed through the sterile pass box before being carried out for further operations. Another trash bin was dedicated to non-infectious materials. Full containers were securely closed and awaited transport in the accompanying vehicle before being sent to the IPD for incineration.

## Discussion

The rising frequency of emerging infectious diseases, their increasing geographic spread and their expanding impact should make overcoming pandemic diseases an international priority. It is widely recognised that infectious diseases constitute social, economic and global security threats.

Outbreak control that relies exclusively on international response, mostly mounted in crisis mode, leaves little time or opportunity to train local teams and is not sustainable, as after the crisis response very little infrastructure or expertise is left behind. If time is of the essence, it seems logical to have local, regional and continental rapid response teams that are well trained and equipped with the appropriate knowledge and tools. Diagnostic needs for pathogens with epidemic potential need to be addressed ahead of the next epidemic. By doing so, we can create a preparedness ecosystem that will allow the shift from a cumbersome, costly emergency response to rapid, cost-effective action for both known and unknown pathogens. Key issues seem to be support for public health emergency preparedness, surveillance and response management, workforce development and knowledge at the regional and country level to withstand emerging diseases and other unexpected health events.

Different models of truck-based laboratories have already been developed for outbreak management or bioterrorism response^17,18,19^; some were deployed during the 2014−2016 Ebola outbreak in West Africa.^19^ However, mobility and flexibility to move from one hotspot to another and energy management, especially in remote areas, turned out to be the biggest challenges.

In this study, the performance of the MBS-Lab was evaluated against field conditions over a six-month period. The MBS-Lab can be considered an off-grid solution that addresses field challenges with regard to mobility, deployment, environment and personnel safety, and operator working conditions. Permanent connectivity using cellular and satellite network connections makes the transmission of data and provision of real-time information possible.

The design of the MBS-Lab offers safe and comfortable working conditions for operators. Designed for and tested by African experts, it has demonstrated successful local ownership and management. Placing patients, their healthcare providers and local communities at the centre of these activities has contributed to local support from both political and medical authorities, which proved to be key factors for a successful initiative. Acting under the auspices of the Senegalese Ministry of Health and local health authorities, the MBS-Lab was fully integrated and embedded into the local health system to reinforce capacity building. The MBS-Lab can be seen as an extension of a strong local reference laboratory that serves as a base station, providing trained operators with logistical support in terms of laboratory consumables and supplies, waste management and storage of biological samples collected during missions.

The pilot study met the overall objective of cutting down turn-around times for diagnostic testing from (at least) a week to hours, through the decentralisation of testing at the most remote level and on-site multiplex testing using molecular platforms at the sentinel sites that served as satellite health posts. No extensive set-up or installation time is required. Once on-site, the MBS-Lab can be operational within the hour and moves easily between sites, which is useful during an epidemic investigation. By avoiding the need for the transportation of infectious clinical samples to centralised laboratories, the time to generate actionable results and logistical burdens are dramatically reduced. This approach led to better-informed decision-making and improved case management, even of highly mobile populations. Because they relied on accurate diagnosis, treatments were more targeted and not based on clinical symptoms only. More than 1300 samples have been safely handled inside the MBS-Lab, including blood samples and nasopharyngeal swabs for the detection of pathogens associated with the main tropical infectious diseases.

Overall, tests were correctly conducted and the results reported on average within 4 h upon receipt. Prior to the deployment, no local laboratory capacity was available in selected sites and samples had to be sent to the IPD for analysis, resulting in a turn-around time of at least a week. In other words, a functional mechanism was in place but might be improved by mobile testing capacity. During the six-month period (from October 2017 to March 2018), acute respiratory infections were the common causes of febrile illnesses in practically all the settings in which the MBS-Lab operated. Human metapneumovirus influenza viruses, parainfluenza virus and picornavirus were the most commonly detected pathogens and were found mostly in children under 5 years, as previously reported.^20^ These results highlighted once again the pivotal role of respiratory viruses in acute respiratory infections. Similar results were found in other countries.^20,21,22^ Co-infection cases have become more apparent since the introduction of multiplex molecular assays; however, the impact on disease severity with arboviruses seems less well defined.^23^ Besides the acute respiratory infections, malaria was also observed in a significant proportion. Malaria is endemic in Senegal, with a stratified transmission pattern characterised by a low incidence in the dry and northern regions and a relatively high incidence in the southern and humid areas. The high transmission season starts from July and continues until October, corresponding to the rainy season.^24^

The majority of malaria cases were found in Kédougou with a peak of transmission in October during the rainy season. These findings are in agreement with other malaria reports, which shows the accuracy of the results provided by the MBS-Lab and indicates that this platform could also be used to monitor the most deadly human parasite.^24,25^ However, no arbovirus cases were observed during the investigation at Kédougou, an area where arbovirus infection is reputed to be endemic.^25^ Whereas in Asia and the Americas human-to-human transmission by mosquitoes is the current form of arbovirus circulation, in West Africa sylvatic circulation is predominant.^26^ With entomological and virological surveillance programmes, several epidemic events have been observed in Kédougou after sylvatic amplification in mosquitoes.^26^ With climate change, urbanisation and population mobility, sporadic cases or small outbreaks are at risk of turning into large epidemics.^27,28^ Indeed, ancestral sylvatic dengue transmission, initially carried by non-human primates and *Aedes* mosquitoes in the forests of West Africa, is now characterised by larger outbreaks, as exemplified by the recent epidemics in Senegal in 2017 and 2018.^29,30^ In that framework, the MBS-Lab was deployed, and 882 (in 2017) and 1736 (in 2018) sera samples were handled (unpublished data). The MBS-Lab played a key role in managing the outbreaks with proximity, rapid response and early patient management.

The response to the dengue virus outbreak has shown that the surveillance network (Senegalese syndromic sentinel surveillance network) can mobilise targeted diagnostic efforts to assist in controlling disease outbreaks. As such, the MBS-Lab complements this system by adding mobile testing capacity.

On top of acute disease surveillance with a direct impact on public health, the MBS-Lab can act in a rapid response capacity to address epidemics, ensure preventive disease surveillance and serve as a health monitoring platform and a provider of primary healthcare. As an open mobile healthcare platform, it offers various opportunities for field-deployable point-of-care technologies (e.g. molecular detection platform with multiplexing or syndromic panel testing, lateral flow, sequencing, etc.).

Moreover, this platform generates high-quality data that can be turned into new insights and knowledge (disease intelligence) and offers great potential for the development of disease surveillance software and epidemiological tools for integrated public health surveillance.

### Conclusion

In summary, we describe the set-up and operations of the MBS-Lab, first deployed in Senegal for extensive field evaluation resulting from a strong partnership between the Praesens Foundation and the IPD. The trained teams and MBS-Lab stationed in Dakar now act as a standby epidemic task force.

Overall, the MBS-Lab is a forward-looking solution for outbreak response in remote areas with a high risk for emerging infectious diseases. However, it can also be considered an open mobile healthcare platform that offers various opportunities for field-deployable point-of-care technologies for surveillance programmes.

It offers rapidly deployable, connected and state-of-the-art technology for effective field diagnostics capabilities. With extensive training and knowledge sharing, this experience perfectly illustrates that by investing in local capacity building efforts that engage communities and establish the necessary trust before a crisis hits, a country will be able to take local ownership of potential future outbreak responses and to address regional laboratory testing needs autonomously. This innovative solution has the potential to be scaled across the African continent. Other domains such as public health emergencies, research, refugee camps, armies, clinical trials and vaccination campaigns also need to be explored for intervention.
